# Nitriding Phenomena in Titanium and the 6Al-4V Titanium Alloy

**DOI:** 10.6028/jres.064A.010

**Published:** 1960-02-01

**Authors:** J. R. Cuthill, W. D. Hayes, R. E. Seebold

## Abstract

Nitriding unalloyed titanium in purified nitrogen at 1,800° F produced a uniformly thick case that consisted of five distinct zones. The same treatment applied to a 6Al-4V titanium alloy resulted in a thinner nitride case that consisted of three zones and elongated nitride grains that penetrated into the core at approximately 45 degrees to the specimen surface. The aluminum appears to be responsible for the formation of the elongated grains. These grains, in turn, appear to be responsible for the nitriding having a more adverse effect on the toughness of the alloy than of the unalloyed titanium, as indicated by preliminary impact test results. The nitride case on the titanium appears to increase in thickness with increase in nitriding time without limit. The nitride case exhibits a hardness equivalent to about 77 Rockwell C at the surface down to almost 50 Rockwell C at the interface.

## 1. Introduction

Titanium is known to have a strong affinity for nitrogen and to form a nitride case of high hardness when heated in the presence of nitrogen or ammonia. However, there has been little previous investigation of the effect of metallic alloying elements on the morphology of the nitride phase or phases generated in the course of the nitriding treatment. This paper is concerned primarily with the effect of aluminum and vanadium. The morphology of the nitride case produced in commercially pure titanium is compared with the nitride formations produced in a titanium 6Al-4V alloy. The work was carried out in the course of a broader program of materials investigation for the Navy Bureau of Aeronautics regarding an application where good resistance to galling and wear between surfaces in sliding contact is required, as well as high surface hardness, light weight, good salt water corrosion resistance, and good machinability. Wyatt and Grant’s [1]^2^ experiments indicated that the nitriding of titanium did produce a surface with satisfactory properties required for the maintenance of surfaces in sliding contact.

Many procedures for surface hardening titanium have been tried by previous investigators, including carburizing, nitriding, and anodizing [2, 3, 4, 5]. It has been generally concluded that nitriding is the most promising method for producing a hard, wear-resistant surface [6]. There has been somewhat less agreement as to the best type of nitriding procedure to use. Wyatt and Grant report a preference for nitriding in ammonia because they found the nitriding time-and-temperature to be lower than that required when using purified nitrogen to produce the same case thickness [4]. On the other hand, some investigators are of the opinion that hydrogen embrittlement of the titanium may be induced during nitriding in ammonia [6]. Therefore, in the initial phase of this current program, it was decided to nitride in purified nitrogen in order to avoid any potential complications involving hydrogen embrittlement.

## 2. Materials

The Armour Research Foundation [5] investigated the effect of various alloy additions on the nitriding characteristics of titanium, and reported that vanadium and boron were the only elements found to result in an increased nitriding rate and increased ultimate hardness. The 6Al-4V alloy, which recently has come into widespread use, therefore, appeared to be of sufficient theoretical as well as practical interest to warrant inclusion in the program along with commercially pure titanium.

The chemical compositions and tensile properties of both materials, as furnished by the supplier, are given in table 1. The material was specified as double arc melted “aircraft quality” 0.050-in. thick sheet, No. 2 finish.

## 3. Experimental Procedures

Specimens, approximately 916 in. by 2½ in., were cut from the 0.050-in. thick sheet; all of the specimens of each material were cut from the same sheet. The edges were milled so as to permit the accurate determination of the cross section of specimens used for unnotched Charpy-type impact tests. Preparatory to nitriding, the specimens were pickled for 15 min in the following standard pickling solution [7] at 80° F:
HNO_3_47 parts by volumeHF2 parts by volumeH_2_O51 parts by volume

Upon removal from the pickling solution the specimens were thoroughly washed in tap water and air dried.

**Figure 1:**
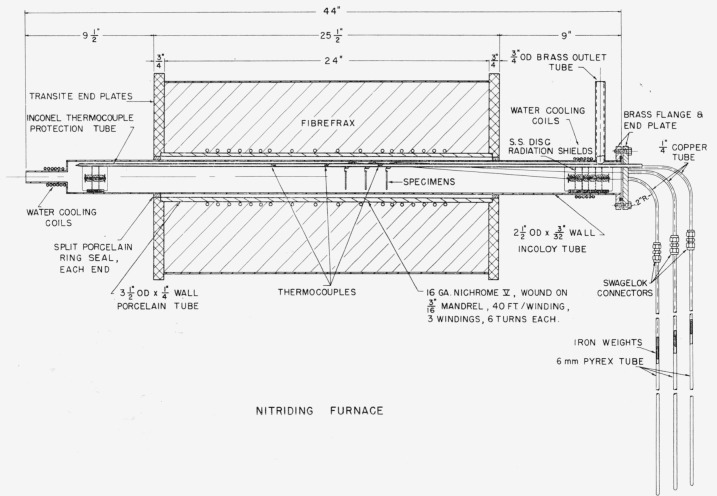
Schematic diagram of apparatus for nitriding specimens for different lengths of time without opening chamber.

Specimens were nitrided at 1,800° F for 4, 16, 24, 48, 96, or 168 hr, to obtain data on case thickness versus time. Figure 1 is a drawing of the nitriding furnace. The nitriding chamber was an Inconel tube with water-cooled ends. The specimens were held in the cool zone at the rear of the nitriding chamber until the chamber with its purified nitrogen atmosphere was brought up to temperature. Each specimen was then individually drawn into the hot zone by magnetic attraction acting on the iron bob attached to the specimen by means of a fine wire. Each specimen, at the end of its prescribed time, was drawn into the cool zone at the front of the chamber by the same mechanism.

“Water-pumped” grade nitrogen was passed through a liquid oxygen cold trap to remove water and then over titanium turnings at 1,800° F to remove oxygen before entering the nitriding chamber. In spite of its strong affinity for nitrogen, titanium was used in the getter furnace because of its superiority in removing oxygen. Fortunately, the oxide reaction is more rapid than the nitride reaction, but, more important, the titanium nitride that is formed eventually reverts to the oxide. However, to insure high efficiency in oxygen removal, the charge of titanium turnings was replaced before every run.

Gas leaving the nitriding chamber was passed through a mercury bubble trap and then exhausted through a wet-test gas meter, which permitted the absolute pressure in the nitriding chamber to be maintained anywhere between 1 and 2 atm. However, tests early in the program revealed no significant difference in nitriding characteristics resulting from varying the pressure over this entire range. Therefore, the bulk of the specimens were nitrided with a pressure of 2 in. of mercury above atmospheric in the nitriding chamber.

More than 300 specimens were nitrided for 48 hr in the course of this program. To facilitate nitriding of this many specimens a much larger chamber was also used. This larger chamber was also of Inconel and the same type of gas purifying system was used, but of correspondingly larger capacity. The specimens were sealed in the chamber, and, with the purified nitrogen flowing through the system, the chamber was then rolled into the furnace after the latter was up to temperature. At the end of the prescribed time, the chamber was rolled out of the furnace and allowed to cool to room temperature before the atmosphere was turned off and the chamber opened.

## 4. Results and Discussion

### 4.1. Microstructure

**Figure 2:**
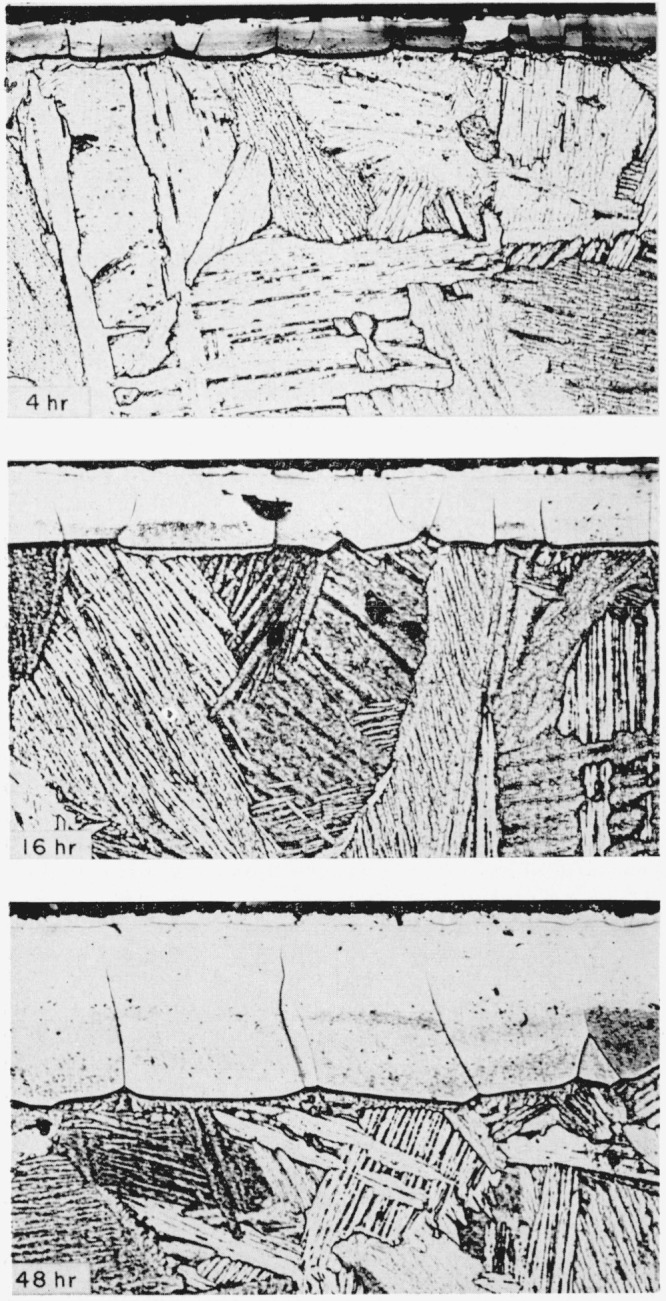
Titanium nitrided in purified nitrogen at 1,800° F for the lengths of time indicated Etched in 1 part HF (48%), 12 parts HNO_3_ (conc), and 87 parts H_2_O. × 200

**Figure 3:**
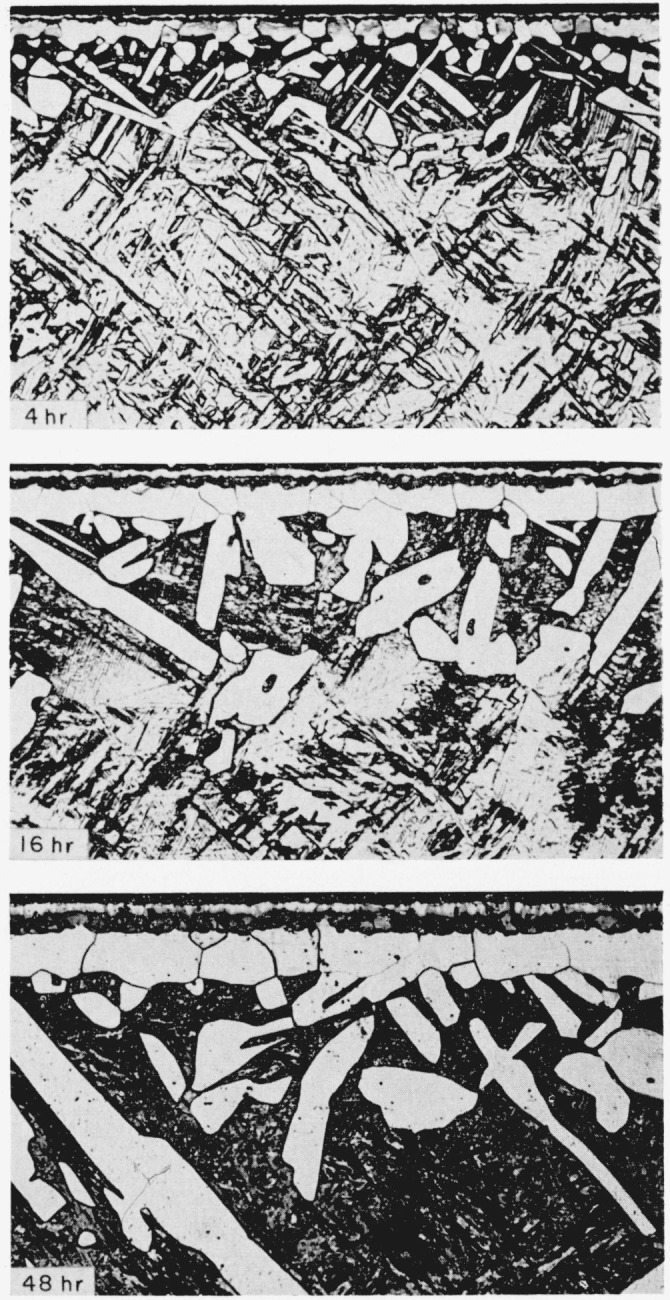
6Al-4V titanium alloy nitrided in purified nitrogen at 1,800° F for the lengths of time indicated Same etchant as for figure 2. × 200.

The typical structures obtained in titanium and in 6Al-4V titanium alloy by nitriding in purified nitrogen are shown in figures 2 and 3, respectively. The immediately evident difference between the metal and the alloy, considering only the gross characteristics, is that the metal forms a uniformly thick case with a sharp line of demarcation between the case and the core, whereas the alloy exhibits elongated nitride grains penetrating as far as the center of the core, given sufficient time, and with the long axes at approximately 45 deg to the surface. These elongated grains are below the uniformly thick case but frequently exhibit apparently common grain boundaries with nitride grains of the surface layer. Also, many of these grains show evidence of having boundaries coherent with the cube planes of the matrix. These elongated grains penetrating into the core might be expected to act as stress raisers and result in lower impact values. Preliminary results do indicate that nitriding has a more adverse effect on the impact properties of the alloy than of the unalloyed titanium.

The hardness of the surface film on both materials was equivalent to about Rockwell C 77, and decreased to approximately C 50 at the interface between the case and the core. The elongated grains in the alloy also exhibited hardnesses equivalent to about Rock-well C 50, in contrast to only about C 35 in the adjacent matrix.

**Figure 4:**
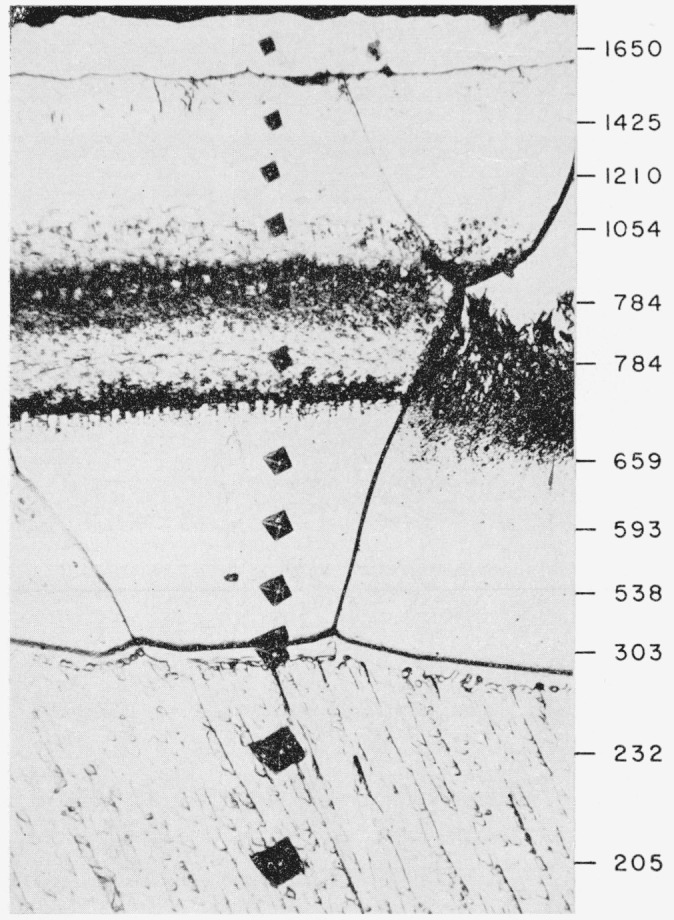
Microstructure and Vickers microhardness indentation (50-g load), and corresponding Vickers hardness numbers, across the case on a titanium specimen nitrided 168 hr at 1,800° F in purified nitrogen Hardness range corresponds to Rockwell C 77 to Rockwell C 12. Same etchant as for figure 2. × 350.

Upon closer examination the “uniform case” on the unalloyed titanium (fig. 2) is seen to consist of a surface film covering a thicker or principal layer. This principal layer is approximately 0.004-in. thick and the surface film is about 0.0005-in. thick on specimens that have been nitrided for 48 hr. There is an abrupt change in etching characteristics at the middle of the layer. Nitriding for a longer time brings out a sharply defined transition band between the upper and lower halves of the layer, as may be seen in figure 4 which also shows a microhardness traverse across the case. The change in etching characteristics is not unexpected because the TiN structure is retained from the stoichiometric composition down to a deficiency in nitrogen corresponding to the composition, TiN_0.42_[8]. It might be mentioned here that to retain the surface film on specimens prepared for metallographic examination, it was necessary to insert sheet plastic between the specimens before placing the pack of specimens in the mounting press for moulding in plastic in the conventional manner. Also, it was preferable that the polishing be done on a wax lap.

**Figure 5:**
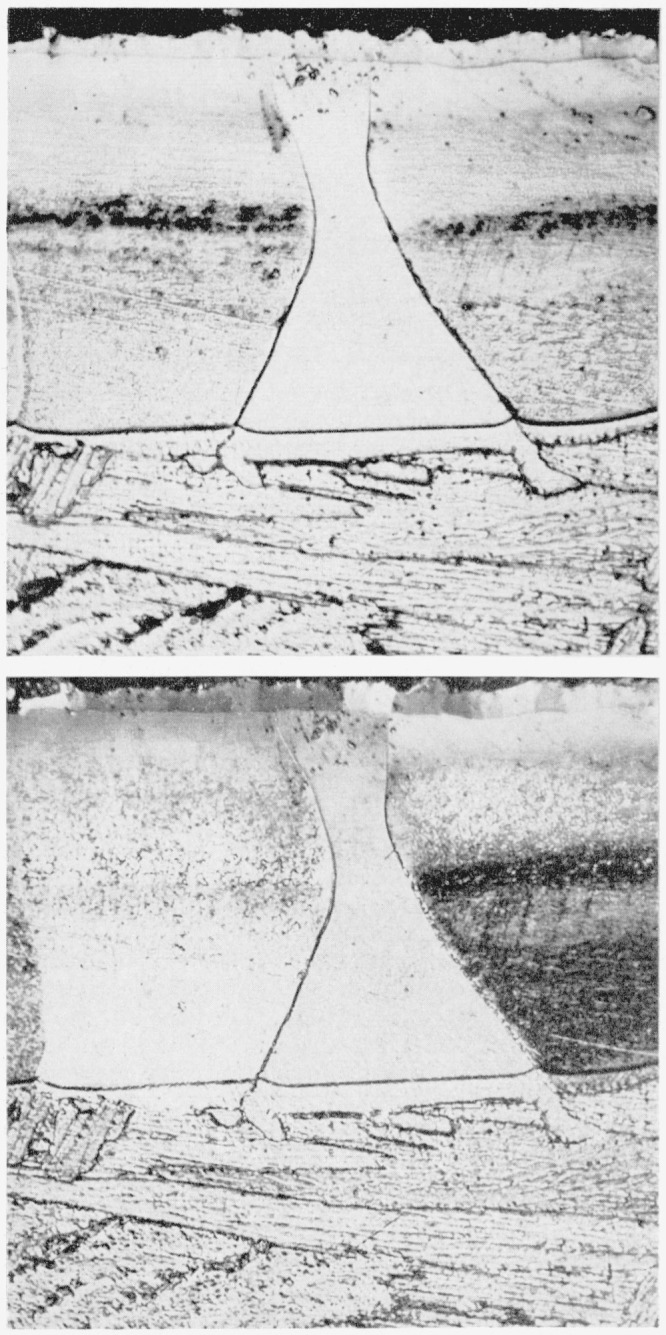
Case and interface between case and core in titanium nitrided 48 hr at 1,800° F in purified nitrogen Same etchant as for figure 2. × 500. Upper: white light. Lower: polarized light.

**Figure 6:**
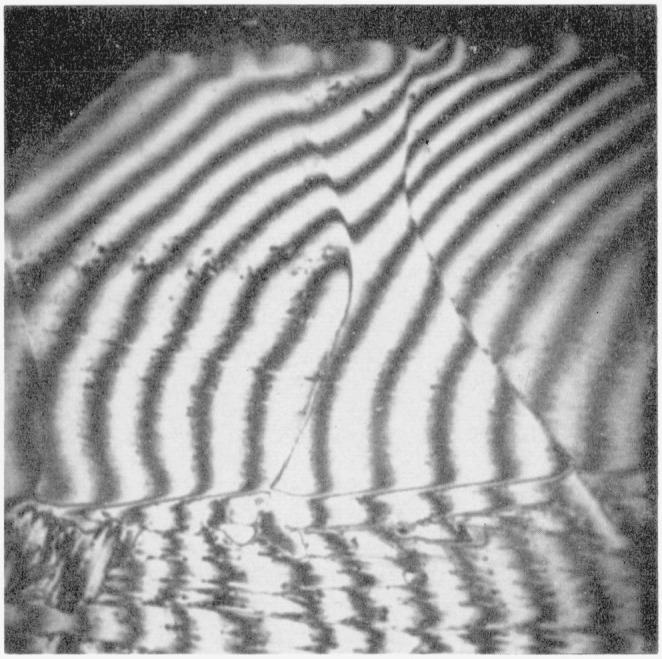
Interference microscope pattern of same specimen as shown in figure 5 One fringe = 11.8×10^−6^ inch difference in elevation.

At the interface between the case and the core of the unalloyed titanium there is a “fringe” of each of the nitride grains of the case protruding over the prominent boundary of the case into the region of the core. That this fringe is actually an integral part of the respective grains of the case is revealed by polarized light, as will be seen by comparing the upper and lower photographs in figure 5. Polarized light (lower photograph) also reveals that the case is composed of grains of different orientations but generally with each grain extending completely through the principal layer of the case and including the “fringe” at the interface with the core. However, the thin surface film is seen to be composed of grains separate from those of the principal layer. Figure 6 is an interference pattern of the same region shown in figure 5, obtained with a Zeiss vertical interferometer microscope. The “fringe” of the “hour glass” shaped grain is revealed to be below the portion of the grain making up the principal layer. Note that the scratches, which are valleys in the specimen surface, provide a means of identifying which are high and which are low areas in the interferometer patterns.

**Figure 7:**
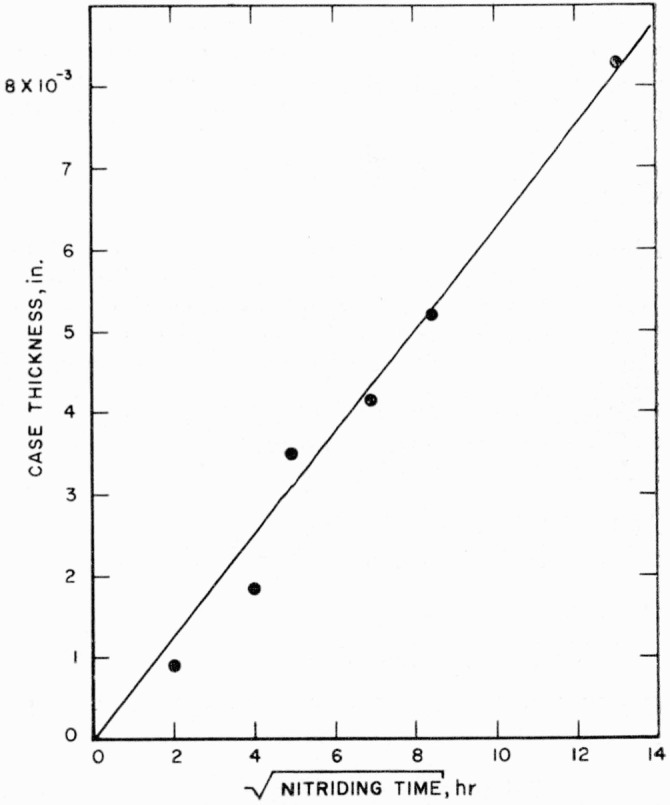
Case thickness in titanium versus square root of nitriding time at 1,800° F.

Wyatt and Grant [3] concluded that a maximum case of approximately 0.004 in. was attained when unalloyed titanium was nitrided in ammonia. The time to produce this maximum was a function of the nitriding temperature. If nitriding were continued beyond the time required to produce the maximum thickness, the case was reported to actually decrease in thickness. They concluded that the mechanism responsible for the decrease in case thickness was the decomposition of the TiN structure at the interface between the case and the core and the nitrogen diffusing into the core at a faster rate than nitrogen diffused through the TiN case to form new case. However, in the present investigation, no evidence for a maximum was found. As shown in figure 7, the thickness of the nitrided case on titanium increased with an increase in time at 1,800° F; a thickness in excess of 0.008 in. was obtained by nitriding 168 hr. Also it is seen that the plotted points fall reasonably well on a straight line, which indicates that the increase in total case thickness with time is a parabolic relationship.

The growth of the elongated nitride grains into the matrix of the 6Al-4V titanium alloy at 45° with the surface in both directions (fig. 3), appears to be associated with the cube planes of a beta phase matrix at 1,800° F. With an electron-probe microanalyzer, vanadium content in these elongated nitride grains was found to be only one-half of that in the matrix. Furthermore, there was no observable vanadium composition gradient in the matrix in the immediate vicinity of the nitride grains. Determinations were made on 18 of the elongated nitride grains in a specimen which had been nitrided for 48 hr. On a sample of pure vanadium, 480 counts per second were recorded, and on a sample of pure titanium 66 cps were recorded when the Geiger counter was set on the V*_Kα_* wavelength. Correcting for this background, 22 to 24 cps were obtained recording the V*_Kα_* wavelength on the nitride grains and 44 to 48 cps on the matrix.

The growth of these elongated nitride grains is obviously a function of nitriding time. Nitrogen and aluminum are strong alpha stabilizers in titanium but vanadium is a strong beta stabilizer. The following calculation of equilibrium constants suggests that the aluminum plays a prominent role in the formation of the elongated nitride grains.

From Kubaschewski and Evans [9], the heats of formation, Δ*F*, are:

$$\begin{array}{l} 2V+N_{2}\to 2(\text{VN})\;\Delta F\\ \;=-83,300+39.7\;T\;\text{cal}/\text{mole}\\ \;=-33,500\;\text{cal}/\text{mole at}\;982{}^{\circ}C\;(1,800{}^{\circ}\;F), \end{array}$$ (a)

$$\begin{array}{l} 2\text{Al}+N_{2}\to 2(\text{AlN})\;\Delta F\\ \;=-121,000+44.5\;T\\ \;=-65,200\;\text{cal}/\text{mole at}\;982{}^{\circ}C\;(1,800{}^{\circ}\;F), \end{array}$$ (b)

$$\begin{array}{l} 2\text{Ti}+N_{2}\to 2(\text{TiN})\;\Delta F\\ \;=-160,500+44.4\;T\\ \;=-104,750\;\text{cal}/\text{mole at}\;982{}^{\circ}C\;(1,800{}^{\circ}\;F). \end{array}$$ (c)

However, to give a more realistic comparison, the data for aluminum and vanadium should be corrected for a 4 and a 6 percent solution, respectively, of aluminum and vanadium in titanium. As a “best approximation,” in lieu of the necessary experimental data on these systems, an ideal solution is assumed and the free energy of dissolution for the corresponding atomic percent concentrations, i.e., 10.2 atomic percent aluminum in titanium, and 3.8 atomic percent vanadium in titanium, is computed from the relationship

$$\Delta F^{M}=RT\;\ln\;N$$

where,

Δ*F^M^* = the free energy of mixing cal/mole of solute. The expression on the right is really the entropy of mixing but because the heat of solution is zero for an ideal solution, the entropy term constitutes the entire free energy.
*R* = the gas constant, 1.987 cal/mole/deg
*T* = the absolute temperature, ° K
*N* = mole fraction of solute

At 1,800° F, one obtains for,

$$\text{Al}\to {\left[10.2\;a/o\;\text{Al}\right]}_{\text{Ti}},\;\Delta F=-5,700\;\text{cal}/\text{mole}$$

and for,

$$V\to {\left[3.8\;a/\text{o V}\right]}_{\text{Ti}},\;\Delta F=-8,200\;\text{cal}/\text{mole}.$$

Combining these results with the values given for the original reactions, (a) and (b), gives

$$2{\left[V\right]}_{\text{Ti}}+N_{2}\to 2(\text{VN}),\;\Delta F=-17,100$$ (a′)

$$2{\left[\text{Al}\right]}_{\text{Ti}}+N_{2}\to 2(\text{Al}),\;\Delta F=-53,800.$$ (b′)

Using the relationship between the free energy, Δ*F*, and the equilibrium constant, *K*,

$$\log\;K=-\frac{\Delta F}{2.30\;RT}.$$

The equilibrium constants for the formation of VN, AlN, and TiN, at 1,800° F, are,

$$\begin{array}{l} \log\;K_{\text{VN}}=+\frac{17,100}{2.303\;RT}=3.05\\ \;K_{\text{VN}}=1\times 10^{3}\\ \log K_{\text{AlN}}=+\frac{53,800}{2.303\;RT}=9.37\\ \;K_{\text{AlN}}=2.4\times 10^{9}\\ \log K_{\text{TiN}}=+\frac{104,750}{2.303\;RT}=18.22\\ \;K_{\text{TiN}}=1.7\times 10^{18} \end{array}$$

These results imply that the nitrogen would combine preferentially with the aluminum in respect to vanadium, and support the experimental result that the aluminum must be largely responsible for the development of the elongated nitride grains which do not occur in the absence of both the aluminum and the vanadium. Furthermore, both the thermodynamic calculations and the electron probe results indicate that the vanadium is not responsible for the formation of these nitride grains. Obviously, a determination of the aluminum content of the elongated grains is needed to confirm or refute this conclusion. Such a determination will be made when a vacuum spectrometer-equipped electron probe, now under construction, becomes available.

### 4.2. X-Ray Diffraction Results

X-ray diffraction patterns were obtained of the surface of the titanium and 6Al-4V alloy before and after nitriding, and again after the nitride surface layer had been polished off.

Un-nitrided titanium exhibited the expected alpha titanium pattern except that the (002) reflection was equally as intense as the (011), the most intense line as reported in the ASTM Index. This is probably due to preferred orientation as a result of rolling. The pattern from the nitrided surface revealed the expected TiN pattern, except that the most intense line was also the (002) reflection instead of the (220) reflection as given in the ASTM Index, and also an extra line corresponding to a “*d*” spacing of 1.52 A. This was the most intense line in the diagram. After polishing off most of the nitride layer, this extra line had not diminished in intensity although the TiN had been reduced drastically and the alpha titanium pattern was beginning to appear.

The alloy, before nitriding, exhibited the expected titanium “*d*” spacings, but the relative intensities differ appreciably from those normally expected from the pure alpha titanium pattern. There was no indication of beta phase. The extra line corresponding to a “*d*” spacing of 1.52 A occurred also in the pattern of the nitrided alloy surface and in the pattern of the alloy after the nitrided surface had been polished off.

## 5. Summary and Conclusions

Nitriding of unalloyed titanium in purified nitrogen produced a sharply defined and uniformly thick case which increased in thickness with increase in nitriding time and apparently would continue to increase without limit. Five distinct zones could be identified. There is no evidence of a maximum case thickness being reached followed by a decrease in case thickness with continued nitriding, as reported by other investigators who nitrided titanium in ammonia.

The same treatment applied to a 6Al-4V alloy of titanium resulted in a thinner nitride case that consisted of three zones and in elongated nitride grains that penetrated into the core at approximately 45° with the surface of the specimen in both directions. The aluminum is indicated to be primarily responsible for the formation of the elongated nitride grains.

The elongated nitride grains, penetrating into the core of the alloy at 45 deg to the surface, apparently act as stress raisers causing a significant reduction in the impact strength of the 6Al-4V alloy.

## Figures and Tables

**Table 1:** Composition and properties of titanium and 6Al-4V alloy^a^

		Titanium	6Al-4V alloy
Yield strength:			
Longitudinal	psi	54.400	128.000
Transverse	psi	53.500	128.300
Tensile strength:			
Longitudinal	psi	68.200	131.600
Transverse	psi	07.500	132.900
Elongation:			
Longitudinal	%	27.0	19.0
Transverse	%	27.0	13.0
Composition:			
C	wt %	0.027	0.01
Fe	wt %	.046	.14
Al	wt %	………	6.0
V	wt %	………	4.1
N_2_	wt %	0.020	0.012
H_2_	wt %	.005	.010
Ti	wt %	99.6	89.5

a Data furnished by manufacturer.

## Footnotes

2 Figures in brackets indicate the literature references at the end of this paper.

## References

[1] Wyatt JL, Grant NJ (1954). Nitriding improves titanium properties. Iron Age.

[2] Griest AJ, Moorehead PE, Frost PD, Jackson JH (1954). Surface hardening of titanium by carburizing and induction heat treatment. ASM Trans.

[3] Wyatt JL, Grant NJ (1954). Nitriding of titanium with ammonia. ASM Trans.

[4] Wyatt JL, Grant NJ (1954). Nitriding produces better hard case on titanium. Iron Age.

[5] Hanzel RW, Pulsifer V Surface hardening of titanium with metalloid elements, June 1, 1952, to May 31, 1953 Rept.

[6] White EL, Miller PD, Peoples RS (1956). Antigalling coatings and fabricants for titanium.

[7] (1954). Metals Handbook, 1954 Suppl. Am Soc Metals.

[8] Ehrlich P (1949). Z Anorg Chem.

[9] Kubaschewski O, Evans E LL (1956). Metallurgical Thermochemistry.

